# 578 A Multi-pronged Approach to Improving Communication, Collaboration and Outcomes in the Burn ICU

**DOI:** 10.1093/jbcr/irac012.206

**Published:** 2022-03-23

**Authors:** Tiffany Hockenberry, Stacey Richerbach, Karen Richey, Kevin N Foster

**Affiliations:** Arizona Burn Center Valleywise Health, Phoenix, Arizona; Arizona Burn Center, Phoenix, Arizona; Arizona Burn Center Valleywise Health, Phoenix, Arizona; The Arizona Burn Center Valleywise Health, Phoenix, Arizona

## Abstract

**Introduction:**

Effective communication between members of the burn healthcare team is vital as it impacts both patient outcomes and staff satisfaction. Discontinuity is a consistent barrier to communication. Fragmented communication may occur for a variety of reasons, including delineated shifts for staff, a decreased presence of physicians and allied health professionals at night, and variable levels of collaboration between nursing, therapy, allied health professionals and physicians. The purpose of this quality improvement project (QI) was to evaluate 3 interventions aimed at improving communication, collaboration, and ultimately patient outcomes.

**Methods:**

The 3 interventions included the use of daily goal cards, increased frequency of interdisciplinary bedside rounds from once daily to twice daily and expanded in-house burn team coverage to include a burn-specific Physician Assistant/Nurse Practitioner (PA/NP) staffed in the center from 3:00pm to 11:00 pm. A 12-item survey consisting of a 5-point Likert scale and open-ended questions was distributed to team members to evaluate effectiveness of the interventions. Incidence of code events were evaluated as an indicator of patient outcomes.

**Results:**

There were a total of 29 respondents, including physicians, allied healthcare professionals, therapists, and nurses. Sixty-nine percent of participants felt that the daily goal cards were an effective means of communication however only 45 % strongly agreed/agreed that achievement of daily goals had increased. The expanded coverage by a burn PA/NP was viewed as an improvement to patient care by 90 % of respondents, with 79 % strongly agreeing/agreeing that the expanded coverage prevented errors. When evaluating afternoon rounds 69 % of respondents either strongly agreed/agreed that it improved patient care and 52% found it to prevent errors. Nocturnal code events decreased by 56% over the one-year period following the institution of expanded coverage. There was no added expense associated with any of the three interventions.

**Conclusions:**

While the results of this QI project were overwhelmingly positive, the expanded coverage by a dedicated burn PA or NP was viewed to be the most beneficial change. Temporally, the expanded coverage was also associated with a marked decrease in nocturnal code events. The addition of afternoon rounds was the next most beneficial change noted with positive findings in both effectiveness and improvement of patient care. There was a dichotomy between the effectiveness of the daily goal cards and the accomplishment of the goals that were set forth that needs further exploration.

